# Validity and Reliability of the Korean Version of the Health Information Technology Usability Evaluation Scale: Psychometric Evaluation

**DOI:** 10.2196/28621

**Published:** 2022-01-24

**Authors:** Jisan Lee, Rebecca Schnall

**Affiliations:** 1 Department of Nursing Science College of Life & Health Sciences Hoseo University Asan Republic of Korea; 2 The Research Institute for Basic Sciences Hoseo University Asan Republic of Korea; 3 School of Nursing Columbia University New York, NY United States; 4 Department of Population and Family Health Mailman School of Public Health Columbia University New York, NY United States

**Keywords:** mobile application, menstruation, survey, questionnaire, translations, medical informatics, app, validity, reliability, usability, assessment, mHealth, evaluation

## Abstract

**Background:**

Rigorous development of mobile technologies requires the use of validated instruments to evaluate the usability of these tools, which has become more relevant with the expansion of these technologies. Although various usability evaluation tools have been developed, there are relatively few simple evaluation instruments that have been validated across diseases and languages in mobile health (mHealth) information technology for use in multiple diseases.

**Objective:**

The purpose of this study is to validate the Korean version of the Health Information Technology Usability Evaluation Scale (Korean Health-ITUES) and assess its applicability for different health conditions.

**Methods:**

To develop the Korean Health-ITUES, we used a validation process involving the following 3 steps: (1) customization of the Health-ITUES for menstrual symptoms, (2) translation of the Health-ITUES from English into Korean, and (3) examination of the reliability and validity of the instrument. The translation process adhered to the World Health Organization (WHO) guidelines for translation and back-translation, expert review, and reconciliation.

**Results:**

The Korean Health-ITUES showed reliable internal consistency with Cronbach *α*=.951; meanwhile, factor loadings of the 20 items in the 4 subscales ranged from 0.416 to 0.892.

**Conclusions:**

The Health-ITUES demonstrated reliability and validity for its use in assessing mHealth apps’ usability in young Korean women with menstrual discomfort. Given the strong psychometric properties of this tool in Korean and English and across 2 different health conditions, the Health-ITUES is a valid and reliable instrument for assessing the usability of mHealth apps. The Health-ITUES is also a valid instrument for evaluating mHealth technologies, which are widely used by patients to self-manage their health and by providers to improve health care delivery.

## Introduction

### Background

In the past decade, 1 of the most challenging components of technology development has been to ensure the usability of tools to ensure their quality in use [1,2]. To support use of the technology, usability must be assessed during the development process [3]. Usability is the measure of the quality of a user’s experience when interacting with a system—whether a website, mobile technology, or any user-operated device [4]. In other words, usability refers to how well users can navigate a system to achieve their goals and how satisfied they are with the process. A successful system needs to work for its users, and it needs to work well. However, many mHealth technologies have been made available to the public, with insufficient attention devoted to their design, development, and evaluation [5]. Technologies produced with poor design and inadequate consideration of the needs of their intended users will be difficult to learn, misused, or underutilized and will ultimately fail to accomplish their objectives [6]. For this reason, usability has been widely recognized as a critical consideration in evaluating the efficacy of technologies [7].

Usability is especially critical for mobile technology, which is widely used in health care [8-10]. In fact, there were more than 800,000 mobile health (mHealth) apps in Apple App Store and Google Play Store in 2020. There is continued growth, with about 200 mHealth apps added each day, with some focusing on healthy eating, physical activity, and improved mental health [11-13]. To date, several studies have evaluated the effect of mHealth apps on disease management and prevention [14-17].

Research to evaluate the usability of mHealth apps has been conducted for various health states, such as chronic obstructive pulmonary disease, HIV, obesity, depression, anxiety, dysmenorrhea, and premenstrual syndrome [18-26]. However, to ensure the rigor of mHealth technologies, it is necessary to create tools to evaluate the quality and usability of mHealth apps [27].

The quality of mHealth apps requires understanding the context of their use and ensuring the apps’ usability [28]. Therefore, it is important to develop mHealth tools using rigorous usability evaluation tools. Although there are several mobile app assessment tools, most have a large number of items or have only been validated for a single disease or in a single language [29,30]. Given these limitations, there is a need for instruments that are validated across languages and diseases. This is especially true in South Korea, which has 1 of the highest rates (94%) of smartphone use in the world. This high penetration of smartphones has enabled the rapid integration of mHealth apps [31,32]. However, despite the high usage of mHealth apps in South Korea, there is a dearth of availability of simple instruments to assess their usability.

### The Study

This study sought to translate the Health Information Technology Usability Evaluation Scale (Health-ITUES) from English into Korean and validate its use. The Health-ITUES is a customized questionnaire comprising 20 items and a modified version of the Health Information Technology Usability Evaluation Model (Health-ITUEM) [33]. The English version of the Health-ITUES has been previously validated through exploratory factor analysis (EFA) and confirmatory factor analysis after use by nurses [34] and community-dwelling adults with HIV [35]; however, it has not been translated into other languages and validated. This study translated the Health-ITUES into Korean and validated it in a sample of 244 women who experienced menstrual-related symptoms and used a menstrual tracking app called PINKDIARY.

## Methods

### Sample/Participants

This study was approved by the institutional review board of the Catholic University of Pusan (CUPIRB-2019-003) before the commencement of study activities. Inclusion criteria for this study were unmarried women >20 and <39 years of age who were previously or are currently using the menstrual-tracking app PINKDIARY for more than a month. The app records the highest usage rate in Korea, and as of October 2021, it was ranked sixth in the health and fitness category in Apple App Store. This app is the official app of the Korean Association of Obstetricians and Gynecologists. PINKDIARY is used to track menstruation and premenstrual symptoms. Features of the app include symptom records, doctor consultations, an online community, and a shopping mall for menstrual items (eg, pads, tampons, and menstrual cups). Marriage and age were also inclusion criteria, as these may have affected the participants’ usage of or experience with using the app.

The sample size was set to ensure a minimum number of participants based on the number of items in the instrument. Nunnally [36] recommended a minimum participant ratio of 10 participants:1 survey item. In this study, the target sample size was between 200 and 250 after multiplying the number of questions (20) by 10. This estimate was based on an anticipated attrition rate of about 20%. In the past, the dropout rate in app-related studies was about 20%-50%. Since this study was not an intervention study, the dropout rate was estimated at 20% [37].

Recruitment was conducted through the KakaoTalk (Kakao Corp) messenger and other online communities (eg, Everytime). Potential participants sent a screenshot of the PINKDIARY app to the researcher’s messenger to authenticate their use of the app. The online consent form and questionnaire were developed in Survey Monkey, and the link was sent to participants on the KakaoTalk messenger. After filling in the consent form and questionnaire, a 2000 won (about US $2) online coffee coupon was sent to the participants as a token of appreciation for their time.

### Step 1: Modification of the Health-ITUES

We customized the Health-ITUES, which was previously validated in a sample of persons with HIV, for women with menstrual discomfort [25]. In this study, menstrual discomfort was defined as primary dysmenorrhea and premenstrual syndrome (PMS), which are the most common menstrual discomfort symptoms among women of reproductive age [38,39]. The modified version was reviewed by the senior author of this manuscript, RS.

### Step 2: Korean Health-ITUES

The translation and back-translation followed WHO guidelines [40].

#### Forward Translation

To align with WHO guidelines, all items had to remain unchanged from their original meaning when translated and had to be translated into English by 2 or more translators. In this study, 2 Korea-born nurses who had lived in the United States for more than 5 years and received doctoral degrees in the United States translated the Health-ITUES from Korean into English. The translators then independently translated it from English into Korean. Following the independent translation, the 2 translators discussed the findings during 3 separate meetings until a consensus was reached.

#### Expert Review

The translated Health-ITUES was reviewed and reconfirmed by a bilingual (English/Korean) physician with dual training in medical informatics. The physician was fluent in both English and Korean. He checked whether the translated version applied to Korean-speaking people and revised the items with expressions or cultural differences that could cause different meanings to be conveyed. After the review process, the translation of the Health-ITUES from Korean into English was considered complete.

#### Back-Translation

Back-translation was conducted by a professional translator from an official translation company and a nurse who lived in the United States for over 10 years and was currently enrolled in a PhD program in the United States. The 2 translators translated the tool back into English and focused on culture and concepts rather than word differences, as was done during the translation process. Inconsistencies were reviewed by the first author to produce a back-translated version of the Health-ITUES.

#### Expert Review

The bilingual physician who participated in the first expert review after forward translation reviewed the back-translated version again. In this step, we focused on whether the Korean words before translation and the Korean words that were translated back had the same cultural and conceptual meanings, rather than focusing on whether they were completely identical.

#### Original Author’s Review

After completing the translation and back-translation, the senior author (RS) reviewed the English version of the Health-ITUES and confirmed the content.

### Step 3: Reliability and Validity

#### Pilot Test

According to WHO guidelines, the minimum number of pilot test respondents is 10 and should represent males and females of all ages and socioeconomic groups. However, in our study, due to time constraints and the fact that we reached data saturation after interviewing 5 women, we limited our sample size to only 5 respondents for this component part of the study [41]. Young women who majored in nursing and had previously used the menstrual app (for a minimum of 1 month to a maximum of 5 years) completed the survey items. Following completion of the survey, they provided feedback about the questionnaire’s items through an in-depth interview. During the interview, we asked the participants to justify their responses and whether they encountered any words in the Health-ITUES that were difficult to understand.

#### Reliability and Validity Test

Internal consistency reliability was measured using Cronbach’s alpha and Pearson’s correlation coefficient. Construct validity was analyzed using exploratory factor analysis. Exploratory factor analysis was used to confirm the predicted factor loadings based on the original instrument [42].

#### Questionnaire

The questionnaire comprised the items described in Table 1.

Table 2 shows each item of the Health-ITUES and the Korean Health-ITUES customized for this study (see Multimedia Appendix 1 for the Korean Health-ITUES).

**Table 1:** The questionnaire’s items.

Categories	Items (N=49), n (%)	Item description	Scale
General characteristics	5 (10)	Sex, marital status, age, residential area, job	N/A^a^
Smartphone experience and menstrual app usage	11 (22)	Smartphone type, which features they used most often, how long, how often they used the menstrual app, etc	N/A
Dysmenorrhea	2 (4)	Pain on the first and second days of the menstrual period [23]	Visual analog scale
PMS^b^	11 (22) Subscale: emotion (4 items), water congestion (4 items), pain (2 items), and appetite (1 item)	The changes that participants experience before menstruation (an appetite item added to the shortened Premenstrual Assessment Form) [43]	6-point Likert-type scale with responses ranging from 1 (not at all) to 6 (very severe change). The higher the score, the more severe the symptoms.
Korean Health-ITUES^c^	20 (42) Subscale: impact (3 items), perceived usefulness (9 items), perceived ease of use (5 items), and user control (3 items)	Impact: high expectations for system impact and perceived usefulness as well as performance assessment of tasks through system usage User-system interactions measured	5-point Likert scale from 1 (strongly disagree) to 5 (strongly agree). The higher the scale value, the higher the usability of the technology. The overall Korean Health-ITUES score is the average of all items with the same weight for each item.

^a^N/A: not applicable.

^b^PMS: premenstrual syndrome.

^c^Health-ITUES: Health Information Technology Usability Evaluation Scale.

**Table 2:** Health-ITUES^a^ and Korean Health-ITUES.

Health-ITUES [24]				Korean Health-ITUES	
Number		Item	Number		Item
**Impact**					
	1	I think Mobile Video Information Provider (mVIP)^b^ would be a positive addition for persons with HIV.	1		I think PINKDIARY^c^ would provide positive health outcomes for women with menstrual discomfort.
	2	I think mVIP would improve the quality of life of persons with HIV.	2		I think PINKDIARY would improve the quality of life of women with menstrual discomfort.
	3	mVIP is an important part of meeting my information needs related to symptom self-management.	3		PINKDIARY helps to meet the information needs for the self-management of my menstrual-related symptoms.
**Perceived usefulness**					
	1	Using mVIP makes it easier to self-manage my HIV-related symptoms.	1		Using PINKDIARY makes self-managing my menstrual-related symptoms easy.
	2	Using mVIP enables me to self-manage my HIV-related symptoms more quickly.	2		Using PINKDIARY allows me to manage my menstrual-related symptoms more quickly.
	3	Using mVIP makes it more likely that I can self-manage my HIV-related symptoms.	3		Using PINKDIARY makes self-managing my menstrual-related symptoms better.
	4	Using mVIP is useful for self-management for HIV-related symptoms.	4		Using PINKDIARY is useful for the self-management of my menstrual-related symptoms.
	5	I think mVIP presents a more equitable process for self-management of HIV-related symptoms.	5		I think PINKDIARY provides a more equitable process for the self-management of my menstrual-related symptoms. (Health equity refers to health equality, which means ensuring the optimal level of health for all people, regardless of income and educational level.)
	6	I am satisfied with mVIP for self-management of HIV-related symptoms.	6		I am satisfied with PINKDIARY for the self-management of my menstrual-related symptoms.
	7	I self-manage my HIV-related symptoms in a timely manner because of mVIP.	7		I can self-manage my menstrual-related symptoms in a timely manner thanks to PINKDIARY.
	8	Using mVIP increases my ability to self-manage my HIV-related symptoms.	8		Using PINKDIARY enhances my ability to self-manage my menstrual-related symptoms.
	9	I am able to self-manage my HIV-related symptoms whenever I use mVIP.	9		I can self-manage my menstrual-related symptoms when I use PINKDIARY.
**Perceived ease of use**					
	1	I am comfortable with my ability to use mVIP.	1		I am satisfied with my ability to use PINKDIARY.
	2	Learning to operate mVIP is easy for me.	2		It is easy for me to learn how to operate PINKDIARY.
	3	It is easy for me to become skillful at using mVIP.	3		It was easy for me to become skillful in using PINKDIARY.
	4	I find mVIP easy to use.	4		I find PINKDIARY easy to use.
	5	I can always remember how to log on to and use mVIP.	5		I always remember how to log on to and use PINKDIARY.
**User control**					
	1	mVIP gives error messages that clearly tell me how to fix problems.	1		PINKDIARY provides error messages that clearly explain how to solve problems with PINKDIARY.
	2	Whenever I make a mistake using mVIP, I easily and quickly recover.	2		I can recover quickly and easily whenever I make a mistake while using PINKDIARY.
	3	The information provided by mVIP (eg, online help, on-screen messages, and other documentations) is clear.	3		The information provided by PINKDIARY (eg, online help, screen messages, and other documents) is clear.

^a^Health-ITUES: Health Information Technology Usability Evaluation Scale.

^b^mVIP: HIV self-management app.

^c^PINKDIARY: menstrual app.

### Statistical Analysis

Descriptive statistics were used to analyze the demographic characteristics of the study participants. Reliability and validity were analyzed using Cronbach *α*, Pearson correlation coefficient, and EFA. All analyses used IBM SPSS Statistics 24.0. Two-sided *P* values of <0.05 were considered statistically significant.

### Ethical Considerations

All study participants provided informed consent, and the study design was approved by the appropriate ethics review board.

## Results

### Pilot Test

Five women completed the draft version of the survey and provided the following feedback about the Health-ITUES:

Overall, I think the questionnaire is easy to understand. However, in the case of items that need multiple responses, I would like for such questions to be clearly marked as “requiring multiple responses.”

Interviewee A

I like this online questionnaire because there are appropriate app icon figures with items. However, I need further explanation about Item 8: I cannot understand what ”equitable process” means.

Interviewee B

I did not have any problem with understanding the items in the questionnaire. However, I went to the next page without answering a few questions. If the respondent does not answer all the items, that is, if even one item has not been answered, please set the questionnaire such that it does not move to the next page.

Interviewee C

As a result of this feedback, item 8 was changed. More specifically, further explanation was added to clarify the term “equitable process” as follows: “Health equity refers to health equality, which means ensuring the optimal level of health for all people, regardless of income and educational level.”

### Demographic Characteristics

A total of 244 unmarried female participants completed the Health-ITUES. The participants’ ages ranged from 20 to 36 years (mean=22.45, SD 3.039). Meanwhile, 127 (51.2%) participants majored in a health-related topic, and 117 (47.0%) participants majored in non-health-related topics for their university degrees. Participants resided in the following cities and provinces: Seoul (n=76, 30.5%), Gyeonggi-do (n=61, 24.6%), Chungcheong-do (n=52, 21.7%), Gyeongsang-do (n=48, 19.4%), and Else (7, 2.8%).

### Internal Consistency Reliability

Internal consistency reliability was measured by Cronbach *α*, and the results are presented in Table 3. All items showed good Cronbach *α* values (>0.8) ranging from 0.83 to 0.94 (Table 3). All values were less than 0.95, which demonstrates that there was no redundancy among the items [44]. Internal scale correlation between items ranged from 0.45 to 0.71, indicating moderate to strong correlations. Notably, perceived usefulness was more highly correlated with impact than other subscales.

**Table 3:** Descriptive statistics, internal scale consistency score, and internal scale correlation for Korean Health-ITUES^a^ subscales (N=244).

Subscale	Mean±SD	Impact		Perceived usefulness		Perceived ease of use		User control	
		Cronbach *α*	r	Cronbach *α*	r	Cronbach *α*	r	Cronbach *α*	r
Impact	10.87±2.52	0.84	—^b^	—	—	—	—	—	—
Perceived usefulness	34.51±6.55	—	0.707^c^	0.94	—	—	—	—	—
Perceived ease of use	19.60±4.13	—	0.510^c^	—	0.660^c^	0.91	—	—	—
User control	10.16±2.43		0.446^c^		0.584^c^	—	0.647^c^	0.83	—

^a^Health-ITUES: Health Information Technology Usability Evaluation Scale.

^b^Not applicable.

^c^*P*<.05.

### Construct Validity

#### Exploratory Factor Analysis

EFA was performed to assess the construct validity of the Korean Health-ITUES items to extract potential factors. Results are reported in Table 4. The Kaiser-Meyer-Olkin measure of sampling adequacy (MSA) was 0.942, indicating that the data in this study were suitable for factor analysis. In addition, as a result of the Bartlett sphericity test, the correlation between the Korean Health-ITUES variables was recognized based on the significance level of .05 with x^2^=3929.635 and *P*<.01. Thus, 4 subfactors were extracted.

**Table 4:** Principal axis factoring with varimax rotation.

Item	Commonality	Component			
		Perceived usefulness	Perceived ease of use	Impact	User control
usefulness8	0.805	*0.831^a^*	0.208	0.149	0.223
usefulness9	0.764	*0.811*	0.197	0.209	0.154
usefulness7	0.677	*0.747*	0.212	0.114	0.249
usefulness3	0.694	*0.735*	0.222	0.306	0.105
usefulness4	0.659	*0.722*	0.143	0.316	0.137
usefulness2	0.663	*0.700*	0.175	0.318	0.201
usefulness1	0.728	*0.687*	0.234	0.401	0.200
usefulness6	0.701	*0.678*	0.323	0.314	0.196
easeofuse1	0.715	0.574	*0.537*	0.183	0.251
usefulness5	0.539	*0.416*	0.162	0.410	0.414
easeofuse2	0.888	0.220	*0.892*	0.161	0.136
easeofuse3	0.901	0.251	*0.863*	0.197	0.233
easeofuse4	0.904	0.314	*0.856*	0.171	0.209
easeofuse5	0.597	0.177	*0.579*	0.080	0.474
impact1	0.789	0.303	0.124	*0.798*	0.065
impact2	0.748	0.309	0.119	*0.798*	0.205
impact3	0.708	0.374	0.284	*0.687*	0.126
control1	0.805	0.204	0.115	0.096	*0.861*
control2	0.765	0.214	0.429	0.062	*0.729*
control3	0.692	0.266	0.255	0.288	*0.688*

^a^Italics indicate the number of items corresponding to the component.

## Discussion

### Principal Findings

A major challenge to technology development is ensuring the usability of the tools [1-3]. However, many mHealth tools are currently available with little attention to their usability [5]. Technologies produced with poor design and inadequate consideration of the needs of their intended users are often difficult to use, and the consumers often cannot accomplish their goals, as a result [6]. Therefore, usability has been widely recognized as an important factor in the development of technology [7].

To address the need for understanding the usability of technology, various studies have been conducted [29,30]. However, each assessment tool could not be easily used, because it had too many questions, took a long time to answer due to difficult questions, was developed for specific users, or was developed only in English [23,29,30,45]. Therefore, the Health-ITUES, a simple and verified tool for multiple populations, was chosen for translation and validation. Because the Health-ITUEM, which is the basis of the Health-ITUES, was developed based on several usability models with strong reliability and validity, including the technology acceptance model (TAM) and ISO9241-11 [46-48]. Additionally, the Health-ITUES was validated in a sample of nurses and community-dwelling adults with HIV [33-35].

This study sought to not only translate but also verify the Health-ITUES considering the Korean context. In step 1, the authors modified the Health-ITUES that was used to evaluate an app for adults with HIV to an app for women suffering from menstrual discomfort. In Korea, there are fewer HIV-infected people compared to the United States. Additionally, because of negative views regarding homosexual contact, HIV-infected people are more likely to not disclose themselves [49]. As of October 2021, when searching for HIV/AIDS-related apps in Apple App Store and Google Play Store, it was difficult to find mHealth for patients with HIV in Korean. To obtain sufficient app users for the validation of the translated tool, the Health-ITUES was modified to enable usability evaluation of the menstrual app PINKDIARY for managing dysmenorrhea experienced by 75% of domestic women [38,50].

In step 2, the Health-ITUES was translated based on WHO guidelines [40]. These guidelines have been used to translate various instruments into many languages, such as Japanese and Arabic, and are not limited to Korean [51-53]. The process of translation and adaptation of instruments were as follows: forward translation, back-translation, and cognitive testing. In this study, 3 experts who majored in nursing or medical informatics and 1 professional English/Korean translator participated in the forward translation and back-translation, respectively. The 2 translators independently translated and reconciled any discrepancies after the forward translation following WHO guidelines. This study was further strengthened by the review of the Korean Health-ITUES by 1 of the authors, who validated the Health-ITUES for mHealth technology. This process ensured that the original meanings of the Health-ITUES items were retained.

Another strength of this reliability and validity is our study sample size. The sample size exceeded the number of items, 20 × 10, and the participants live in various regions in Korea. Their field of work (major) is also not biased, so it can be said that geographical biases are small.

Moreover, in step 3, internal scale correlation ranged from 0.45 to 0.71, which indicates moderate to strong correlations. Notably, perceived usefulness was more highly correlated with impact than the other factors. The results mirrored the findings from the validation study using the Health-ITUES in a sample of adults with HIV [34]. In EFA, in the case of values for *usefulness*, 5 items (“I think PINKDIARY provides a more equitable process for the self-management of my menstrual-related symptoms.”) were included as 3 components because the values were so similar: 0.416, 0.410, and 0.414. As a result, we decided to keep this item with the first component *perceived usefulness* with which it is most closely conceptually aligned [54].

### Limitations

One limitation of this study is that when recruiting survey participants, only the experience of using the menstruation-related mHealth app was checked and no restrictions were placed on the past period of use. Future research should recruit participants by suggesting clear past usage periods based on objective evidence. However, this study successfully translated the Health-ITUES from English into Korean and validated it, and this instrument can be used to evaluate the usability of mHealth apps. These findings will contribute to the systematic evaluation of the rapidly growing field of mHealth apps.

### Conclusion

The Health-ITUES demonstrated reliability and validity for use in assessing mHealth apps’ usability in young Korean women with menstrual discomfort. Given the strong psychometric properties of this tool in Korean and English and across 2 different health conditions, the Health-ITUES is a strong instrument for evaluating the usability of mHealth apps.

## Appendix

Multimedia Appendix 1 The Korean version of the Health-ITUES. Health-ITUES: Health Information Technology Usability Evaluation Scale.

## Abbreviations

EFA: exploratory factor analysis
Health-ITUEM: Health Information Technology Usability Evaluation Model
Health-ITUES: Health Information Technology Usability Evaluation Scale
mHealth: mobile health
MSA: measure of sampling adequacy
PMS: premenstrual syndrome
TAM: technology acceptance model
WHO: World Health Organization
