# Supplementary figures and images for: Validity and Reliability of the Korean Version of the Health Information Technology Usability Evaluation Scale: Psychometric Evaluation
Source: JMIR Med Inform. 2022 Jan 24;10(1):e28621. doi: 10.2196/28621 (PMC8822430; doi:10.2196/28621)

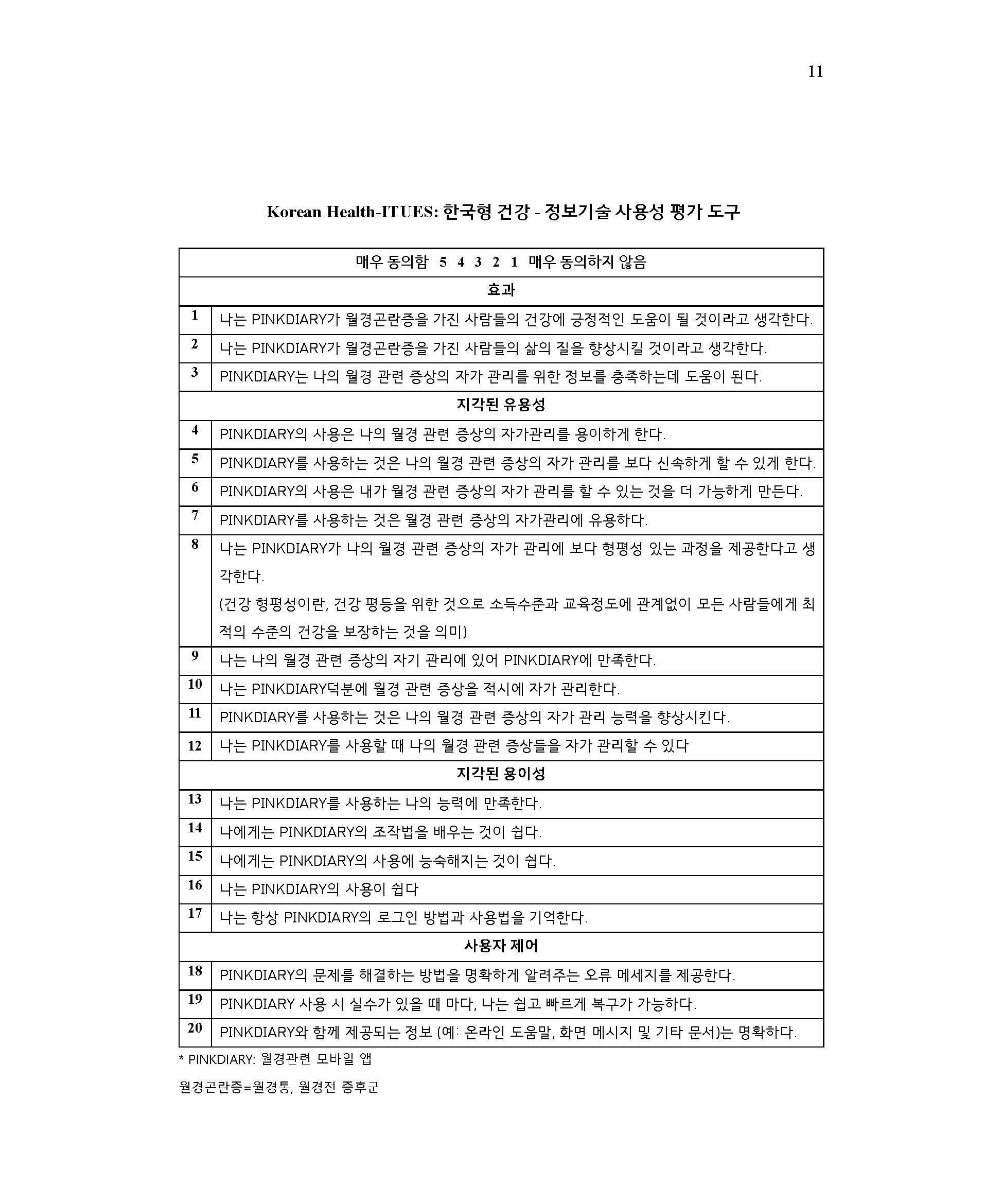

Supplement: Multimedia Appendix 1 [file medinform_v10i1e28621_app1.png]
